# Production and Properties of the Porous Layer Obtained by the Electrochemical Method on the Surface of Austenitic Steel

**DOI:** 10.3390/ma15030949

**Published:** 2022-01-26

**Authors:** Agnieszka Ossowska, Jacek Ryl, Tomasz Sternicki

**Affiliations:** 1 Faculty of Mechanical Engineering and Ship Technology, Gdansk University of Technology, 80-233 Gdańsk, Poland; sternicki.tomasz@gmail.com; 2 Advanced Materials Center, Faculty of Applied Physics and Mathematics, Gdansk University of Technology, 80-233 Gdańsk, Poland; jacek.ryl@pg.edu.pl

**Keywords:** anodization, oxide layer, austenitic stainless steel, porous layer, biomaterial, corrosion resistance

## Abstract

The growing demand for implants has seen increasing interest in the introduction of new technologies and surface modification methods of metal biomaterials. This research aimed to produce and characterize a porous layer grown on austenitic stainless steel 316L, obtained via the anodization process near the micro-arc oxidation, i.e., low voltage micro-arc oxidation (LVMAO). The discussed layer significantly influences the properties of metallic biomedical materials. The surface topography, layer thickness, surface roughness, pore diameter, elemental composition, crystal structure, and surface wettability were assessed for all anodized layers, together with the resultant corrosion resistance. Attention was paid to the influence of the process parameters that affect the specification of the produced layer. The obtained results showed surface development and different sized pores in the modified layers, as well as an increase in corrosion resistance in the Ringer’s solution.

## 1. Introduction

There is currently a growing demand for materials that can replace damaged or weakened bones, joints, muscles, and ligaments. Implanted biomaterials must fulfill several criteria. They must be biocompatible, have long-term endurance to constant and variable stresses, and finally, have corrosion resistance in a physiological environment. A properly selected biomaterial ensures the implant’s long-term stability, functionality, and appropriate biological reactions of living tissues in its vicinity. The properties of the biomaterial mainly determine the durability and functionality of the implant, while they are responsible for the biological reactions on its surface: chemical state, surface topography, roughness, wettability, flexibility, surface energy, etc. [1].

Metal biomaterials, including cobalt-chromium alloys (CoCr), titanium (commercially pure Ti) as well as titanium alloys (e.g., Ti-6Al-4V, Ti13Nb13Zr), and stainless steels (316L), have been used for a long time. Due to their high corrosion resistance, good strength, ductility, and high hardness [2], austenitic steels are successfully used as screws, intramedullary nails, fixation elements, etc. They are characterized by a fine-grained structure and a low level of non-metallic inclusions, ensuring adequate strength and ductility of the steel, and reducing the likelihood of cracks. The chemical composition of austenitic steels determines their single-phase and paramagnetic structure, as well as good resistance to pitting corrosion and good mechanical properties [3].

Apart from the appropriate strength properties, the most important criterion for the use of some stainless steels as biomaterials is their biotolerance, which depends on bioelectric, biochemical, and biomechanical factors initiating bacteriological, metabolic, immunological, and oncological processes. Modern alloys used in implantology should meet the criteria of:

- tissue compatibility (biocompatibility),
- non-toxicity and non-allergic,
- cytotoxic and carcinogenic,
- bioactivity enabling acceleration of the adhesion process with living tissue,
- blood-compatibility,
- no tendency to form clots, which depend on location in the blood stream,
- the contact time of the implant surface with blood,
- the blood flow velocity,
- the chemical composition of the surface [4].

Despite the many advantages of these materials, there are still problems with implant loosening, which may be caused by stress shielding [5,6,7], the action of pitting or crevice corrosion, and possible allergic reactions. In order to avoid such problems and increase biocompatibility, the bioactivities of austenitic steels are subjected to various types of surface modification processes. The basic methods causing changes in surface quality include mechanical processing (grinding, polishing), laser ablation [8,9,10], thermal oxidation [11], chemical oxidation [12,13,14], micro-arc oxidation (MAO) [15], and oxidation with hydrogen peroxide [16] or electrochemical treatment [17,18,19,20,21,22,23]. By modifying the electrolyte composition and current values in the electrochemical oxidation, we can obtain layers of different chemical compositions with different structures, e.g., amorphous, nanotube, and as a result of the application of high voltage treatment, porous layers [24,25]. Another advantage of using electrochemical oxidation is the possibility of doping the layers with various ions, e.g., Au, Zn, or Cu [26,27,28,29].

Different roughness may be obtained when using this type of processing [30,31]. As a result of such surface modification, the abrasive coat [32,33], surface wettability [34,35,36,37,38,39,40,41,42,43,44], adhesion, and biocompatibility [45,46,47,48] are changed. Moreover, the oxide layer may influence the osteoinduction processes by changing the oxides’ architectural features and chemical composition [49].

The great advantage of porous layers produced by micro-arc oxidation is the enhanced corrosion resistance [50,51,52,53], which is an important aspect in the selection of components used for medicine and other industries. Unfortunately, there is little literature on porous layers’ characteristics on stainless steel produced by the micro-arc oxidation.

This research is focused on producing and characterizing anodized porous layers with the goal to enhance the properties of metallic biomedical materials. The application of electrochemical oxidation with high values of electric voltage, close to the value of micro-arc oxidation (LVMAO), made it possible to obtain porous layers with specific pore sizes. Modification of the surface of austenitic 316L steel also improved the corrosion resistance properties.

## 2. Materials and Method

The study was performed on austenitic steel of the grade 316L, with its chemical composition listed in Table 1. The sheet was delivered annealed at 1050 °C.

The specimens of dimensions 15 × 10 mm were cut from the alloy sheet with an initial thickness of 0.7 mm. The samples were ground with abrasive papers (No. 2000 as the last, Struers Inc., Cleveland, OH, USA). Afterward, the specimens were cleaned in an ultrasonic chamber (Sonic-2, Polsonic Palczynski Sp. J., Warszawa, Poland) with isopropanol, methanol (Avantor Performance Materials Poland S.A., Gliwice, Poland), and distilled water, subsequently, for 5 min in each batch, and finally dried in cold air.

The LVMAO was made in ethylene glycol (C_2_H_6_O_2_, pure p.a. 99%, Chempur, Piekary Śląskie, Poland) and perchloric acid (HClO_4,_ 60%, Chempur, Poland). The tests were performed in a standard electrical circuit composed of an electrochemical cell, power supply, Pt electrode as the polarizing electrode, and the tested metallic electrode. Neither stirring, aeration, nor deaeration were applied. All measurements were performed at room temperature. A charging time of 15 min was used with a constant cell voltage of 100 V, 150 V, or 200 V (Table 2).

The specimens’ surfaces and cross-sections after each applied oxidation condition were examined with the scanning electron microscope (SEM JEOL JSM-7600F, JEOL Ltd., Tokyo, Japan), equipped with an LED detector at 5 kV acceleration voltage. The layers’ chemical composition was determined using X-ray energy-dispersive spectroscopy (EDS) (Edax Inc., Mahwah, NJ, USA).

The X-ray diffraction studies were carried out using an X-ray diffractometer (Philips X’Pert Pro–MPD, Brighton, UK) system vertical T-T goniometer (190 mm radius). The X-ray source was a long-fine-focus, ceramic X-ray tube with Cu anode. The standard operating power was 40 kV, 50 mA (2.0 kW). The system optics consisted of programmable divergence, anti-scatter and receiving slits, incident and diffracted beam soller slits, curved graphite diffracted beam monochromator, and a proportional counter detector (Bragg–Brentano parafocusing geometry (2Θ ca. 5–100°).

The X-ray photoelectron spectroscopy (XPS) measurements were carried out using Escalab 250Xi spectroscope (ThermoFisher Scientific, Waltham, MA, USA), operating with a monochromatic AlKα X-ray source, with a spot size of 250 µm. The pass energy was 150 eV for survey spectra and 20 eV for high-resolution spectra recorded in *Ni 2p*, *Fe 2p*, *Cr 2p*, *Mo 3d*, and *O 1s* core-level binding energy ranges. Low-energy electron and low-energy Ar^+^ ion flow were used for charge compensation purposes during the experiment, with the final peak calibration using adventitious *C 1s* peak at 284.6 eV. Spectral deconvolution was performed with Avantage software (v.5.973, ThermoFisher Scientific, Waltham, MA, USA).

The nanoindentation tests were performed with the NanoTest Vantage (Micro Materials, Wrexham, UK) equipment using a Berkovich three-sided pyramidal diamond. The maximum applied force was equal to 5 mN, the loading and unloading times were set at 20 s, the dwell period at full load was 10 s. The distances between the subsequent indents were 50 μm. During the indent, the load–displacement curves were determined using the Oliver and Pharr method. Based on the load–penetration curves, the surface hardness (H) and reduced Young’s modulus (E) were calculated using the integrated software. The critical process parameters included the maximum force, holding time, and test rate. In calculating Young’s modulus (E), a Poisson’s ratio of 0.3 was assumed for the titanium oxide layer. The measurements were processed in randomly selected five points for each surface, and the results were averaged.

The water contact angle (wettability) measurements were taken for the reference stainless steel 316L and oxidized specimens using a contact angle goniometer (Zeiss, Jena, Germany) at room temperature. All the analyses were repeated three times.

The electrochemical impedance spectroscopy (EIS) measurements were carried out in a three-electrode setup, with the studied sample as the working electrode, silver chloride electrode as the reference, and Pt mesh as the counter electrode. The studies were performed using Gamry Reference 600+ potentiostat/galvanostat (Gamry Instruments, Warminster, PA, USA). The applied perturbation signal frequency range was set between 40 kHz and 10 mHz, with a perturbation amplitude of 10 mV. This experiment was carried out at 37 °C, the conditioning period before the experiment lasted for 2 h. Naturally aerated Ringer’s solution was the studied electrolyte and its volume in the corrosion cell was 100 mL.

## 3. Results and Discussion

### 3.1. Microstructure, Surface Topography, Phase, and Chemical Compositions

**Figure 1:**
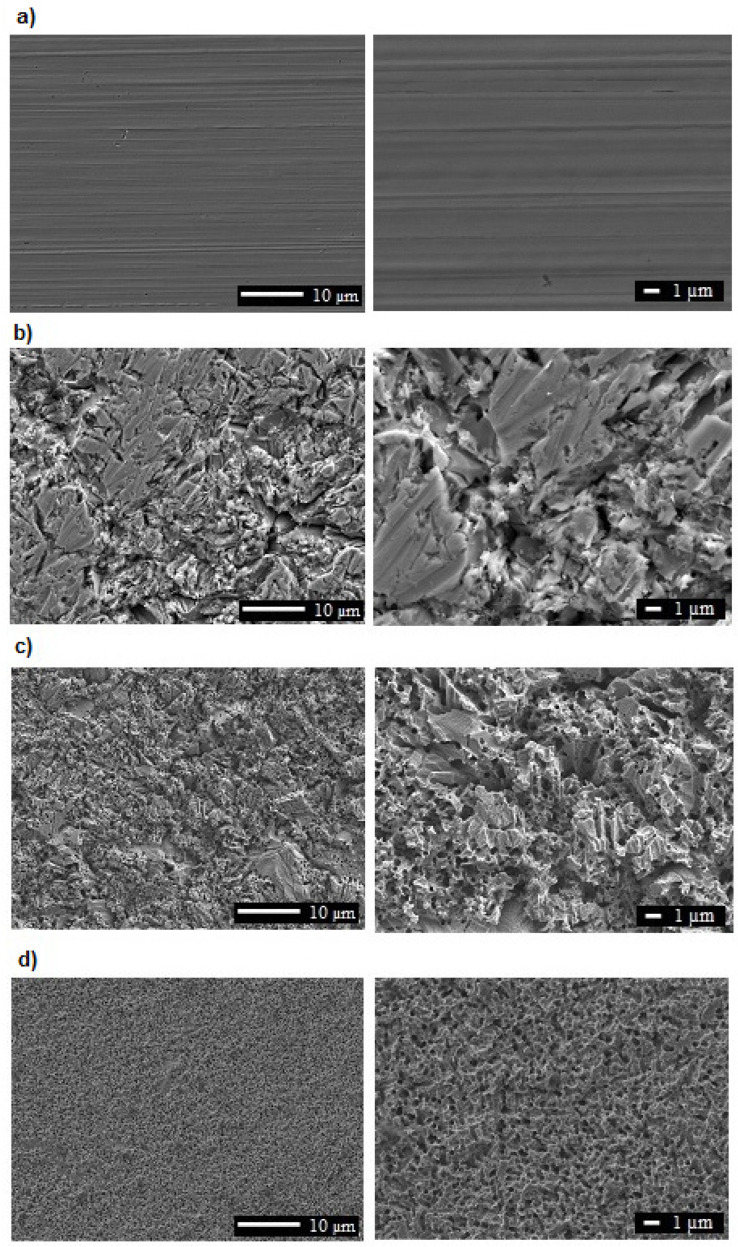
SEM micrographs presenting the morphology of the (**a**) 316L steel and LVMAO various potencials: (**b**) sample I with 100 V, (**c**) sample II with 150 V, (**d**) sample III with 200 V.

**Figure 2:**
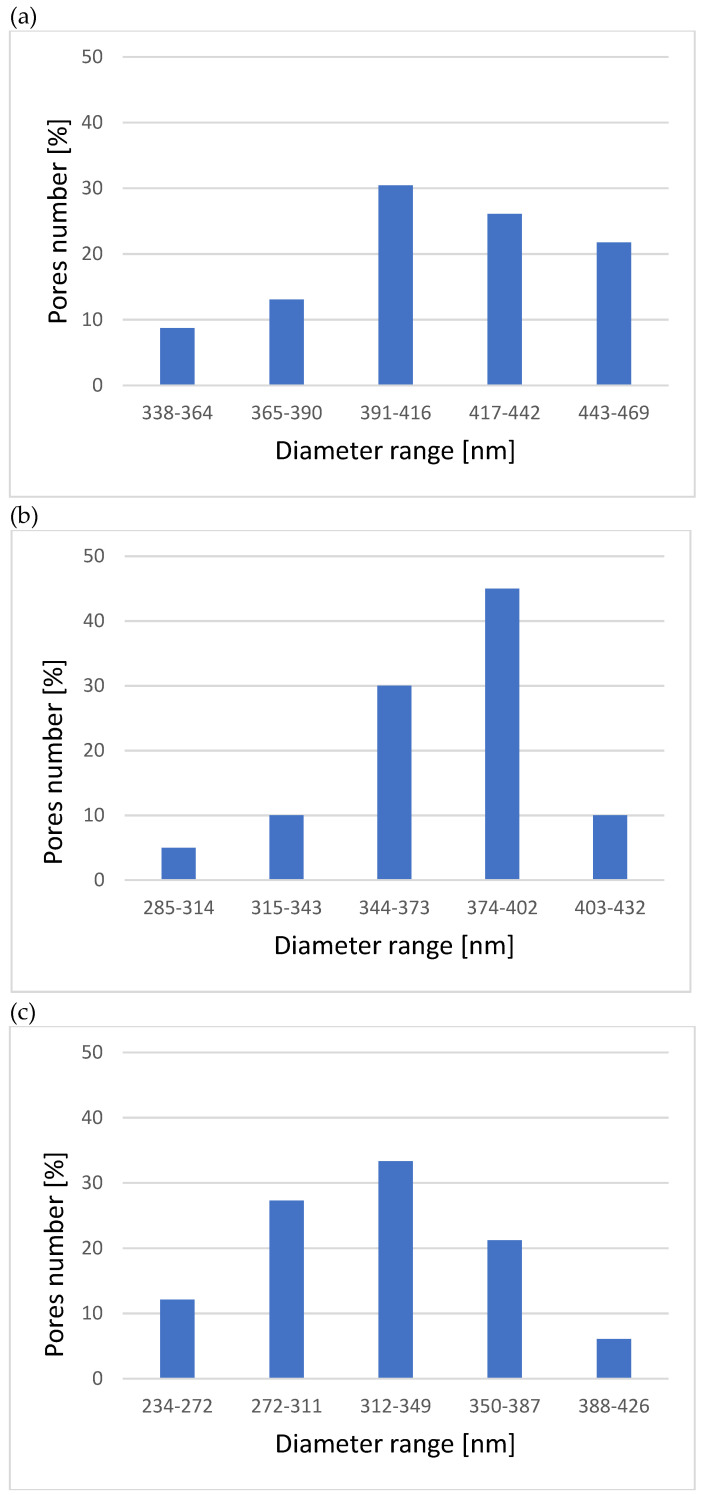
Pore diameters histograms for samples: (**a**) sample I, (**b**) sample II, (**c**) sample III.

Figure 1 presents the morphology of the anodized oxide layers, showing a different structure depending on the process parameters. The layers obtained with the use of the lowest voltage, sample I (100 V), did not uniformly cover the alloy surface (Figure 1b), with visible areas showing an absence of the porous layers. This sample showed only the residuals of the porous layer formation. The gradual increase in the applied voltage made the porous layer cover the samples entirely, as supported by SEM micrographs of samples II and III (Figure 1c,d, respectively). The layers differed not only in the surface morphology, as significant differences were also found in the pore size (Figure 2). For sample I, the produced pores contained the largest proportion of pores with large diameters, from 390 to 469.4 nm. As previously noted, the porous layer did not evenly cover the entire surface of the sample I. There were places on the surface of the samples where the process of formation of the porous layer did not begin. The pore sizes of sample II showed smaller diameters (max. 432 nm), and pores with a diameter of 344–402 nm constituted about 75% of the sample surface. However, for sample III, the maximum diameters of the pores were 426.2 nm. Pores with a diameter of 272–387 nm dominated. The surfaces of sample III showed high homogeneity of the porous layer and a similar size of evenly distributed pores.

**Figure 3:**
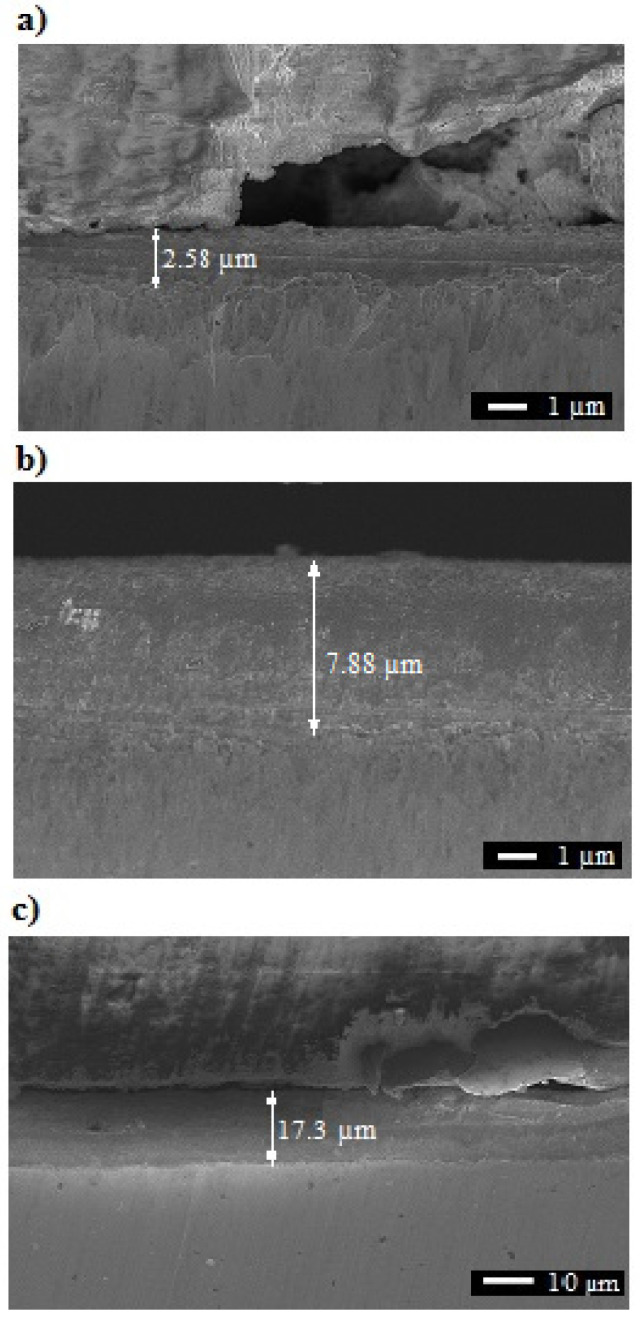
Thickness of the layers: (**a**) sample I, (**b**) sample II, (**c**) sample III.

Due to the increasing values of the voltage, one should expect divergent thicknesses of the obtained layers (Figure 3 and Table 3), which for layers formed at the surface of samples ranged from 2.1 μm (Figure 3a) to 17.5 µm (for sample III, Figure 3c), gradually increasing with the increase of the applied anodization voltage.

The main factor affecting the formation of the LVMAO layer is the electric field, which overcomes the energy barrier associated with the activation of diffusion of ions from the metallic phase to the oxide phase. Ions migrate through the oxide layer towards the oxide–electrolyte interphase. The low electrical conductivity and high value of the layer’s energy barrier require the use of an electric field of 10^−6^–10^−8^ V cm^−1^, which enables the transport of ions during electrochemical oxidation. A suitable distribution of potentials takes place at the phase boundaries: oxide/electrolyte, metal/oxide. As a result of electrochemical oxidation, the oxide layer thickness for titanium alloys is a function of anodizing potential and time. The applied voltage affects the obtained thickness of the oxide layer [54], while the used electrolyte determines the growth rate, which is the result of the oxide layer formation rate and the oxidation product dissolution rate [55]. The thickness of the layer is an important element preventing direct contact of the material with the external environment. This is a type of barrier that may increase corrosion resistance and thus extend the service life of the element protected by a porous layer applied as a result of the LVMAO process.

**Figure 4:**
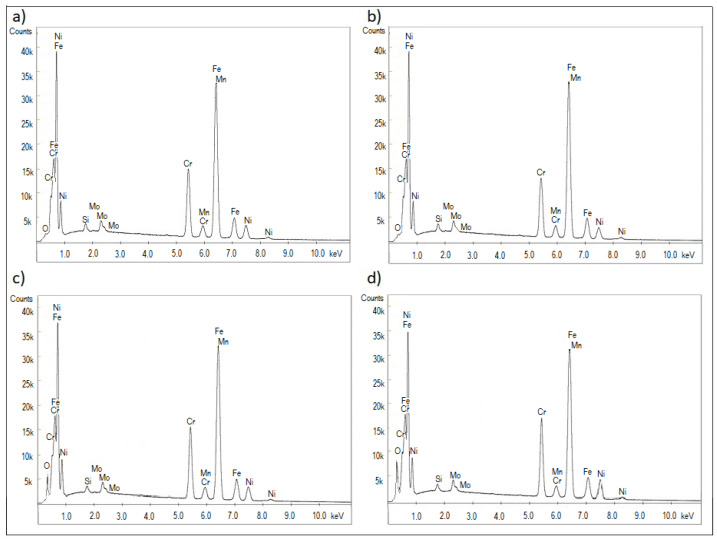
EDS examination of chemical composition of: (**a**) reference, (**b**) sample I, (**c**) sample II, (**d**) sample III.

The results of tests of the chemical composition of the cross-sections of the layers carried out with the use of EDS measurements are presented in Table 4 and Figure 4. In all the produced layers, a decrease in the content of the primary 316L alloy components (Fe, Cr, Ni, Mo and Mn) and increase in the oxygen content were observed. Analyses of the chemical composition of the layers showed that the content of the primary Fe element in the 316L stainless steel drastically decreased with the increase of the layer thickness and the applied tension. A similar relationship was also observed for Mo. However, with the increase in layer thickness and tension, much higher values of Cr and Ni were noted. The increase in the concentration of the elements Cr and Ni can be explained by the higher affinity of Cr and Ni with oxygen compared to the affinity for Fe, for the formation of chromium oxides and nickel oxides.

Similar results were previously obtained for steel 304 [55], which showed that the composition of the porous layers is similar to the composition substrate (parent material). The research [56] revealed that the concentration of H_2_O in the anodic bath determines the morphological features of these anodically formed layers. Research conducted by Tsuchiya [54] showed that the addition of water to the organic baths also increased the pore diameter. On the other hand, Asoh [50] proved that the influence of the electrolyte and the applied voltage leads to porous layers of different chemical compositions.

**Figure 5:**
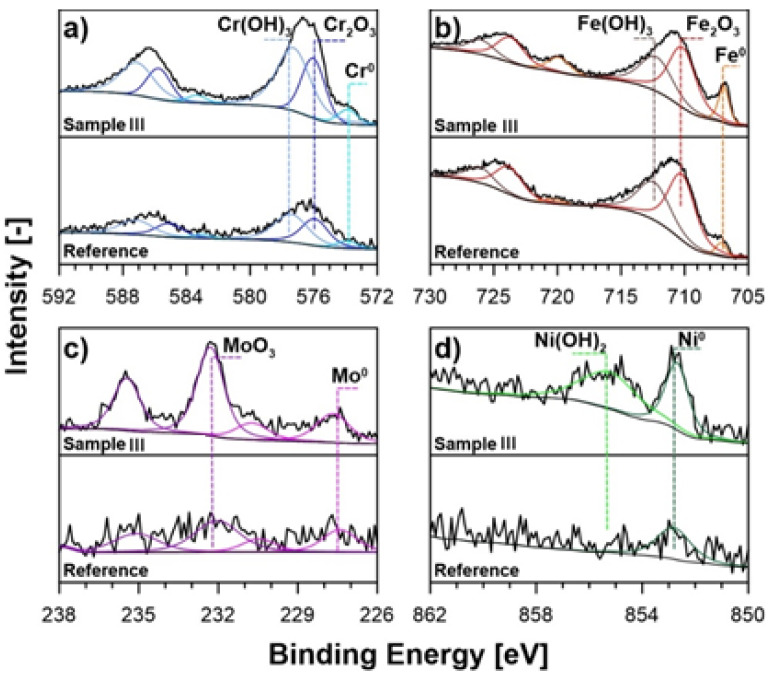
High-resolution XPS spectra for Sample III and reference 316L sample, recorded in the core-level binding energy range of: (**a**) *Cr 2p*, (**b**) *Fe 2p*, (**c**) *Mo 3d* and (**d**) *Ni 2p_3/2_*.

The high-resolution XPS analysis, performed for each studied sample, revealed a considerable difference in each LVMAO-formed layer surface chemistry compared to the reference 316L steel sample. This analysis concerns the chemistry of the native passive oxide film on each studied porous layer’s surface due to the XPS analysis depth. Figure 5 shows the spectra recorded in the binding energy range of *Cr 2p* (Figure 5a), *Fe 2p* (Figure 5b), *Mo 3d* (Figure 5c), and *Ni 2p_3/2_* (Figure 5d).

When considering the reference sample, it may be observed that it is primarily built of iron and chromium compounds. The peak deconvolution of iron *Fe 2p* spectra (Figure 4a) reveals the dominant presence of two peak doublets with *Fe 2p_3/2_* peaks located at 710.3 and 712.3 eV, values typically found for the Fe(III) oxidation state, in the form of oxides and hydroxides, respectively [57,58,59]. However, a small signal from metallic iron with *Fe 2p_3/2_* at 707.0 eV was also found, which attests to the passive film’s thickness in the air atmosphere does not exceed approx. 5 nm. The total share of iron among all the metallic atoms identified on the reference sample’s surface was 69.2 at.%, as shown in Table 5. On the other hand, the share of the primary alloying additive, chromium, reaches 26.5 at.%. This element is primarily found in the form of Cr(III) oxides (*Cr 2p_3/2_* at 576.0 eV) and hydroxides (577.3 eV). However, as with iron, a small contribution from metallic Cr atoms was found [60,61]. Finally, small contributions from MoO_3_, metallic Mo, and metallic Ni were also recognized, as presented in Table 5 [61,62,63].

As a result of the LVMAO porous layer formation, the investigated samples’ surface chemistry changes significantly. The XPS analysis revealed that the share of chromium oxides and hydroxides increased by nearly half and up to 35.5 at.% for Sample III, a layer grown at the polarization voltage of 200 V. A similar observation was made in the case of MoO_3_ contribution, which amount reaches over 4.6 at.% among all the studied elements. Notably, the amount of the Cr(III) and Mo(VI) oxides/hydroxides depends on and rises with the polarization voltage applied during the anodic layer formation. An opposite conclusion may be drawn when analyzing the chemistry of iron. Here, the total share of iron(III) species drops down from 66.9 to 43.9 at.% in the case of Sample III. Notably, the electrochemical formation of the porous layer at the surface of 316L steel samples leads to the appearance of the oxidized form of nickel (see Figure 5d), with an *Ni 2p_3/2_* peak at 855.4 eV that was identified as Ni(OH)_2_ based on the literature survey [62]. Nickel oxides do not typically contribute to the stainless steel passive film [63], while the Ni(OH)_2_ contribution did not depend on the applied polarization voltage.

The overall conclusion to be drawn is that the formation of the porous layer at the surface of the analyzed stainless steel samples increases the nobility of the elements building the native passive film, which should translate to a higher corrosion resistance of the studied material, most importantly for samples II and III. Another interesting conclusion may be drawn based on the total share of peaks corresponding to the metallic atoms at zero oxidation state (Fe^0^, Cr^0^, Mo^0^ and Ni^0^), in that the native passive film at the surface of Samples I–III is thinner than this for the reference sample. The effect, however, might originate from the altered surface geometry introduced by the porous layer.

**Figure 6:**
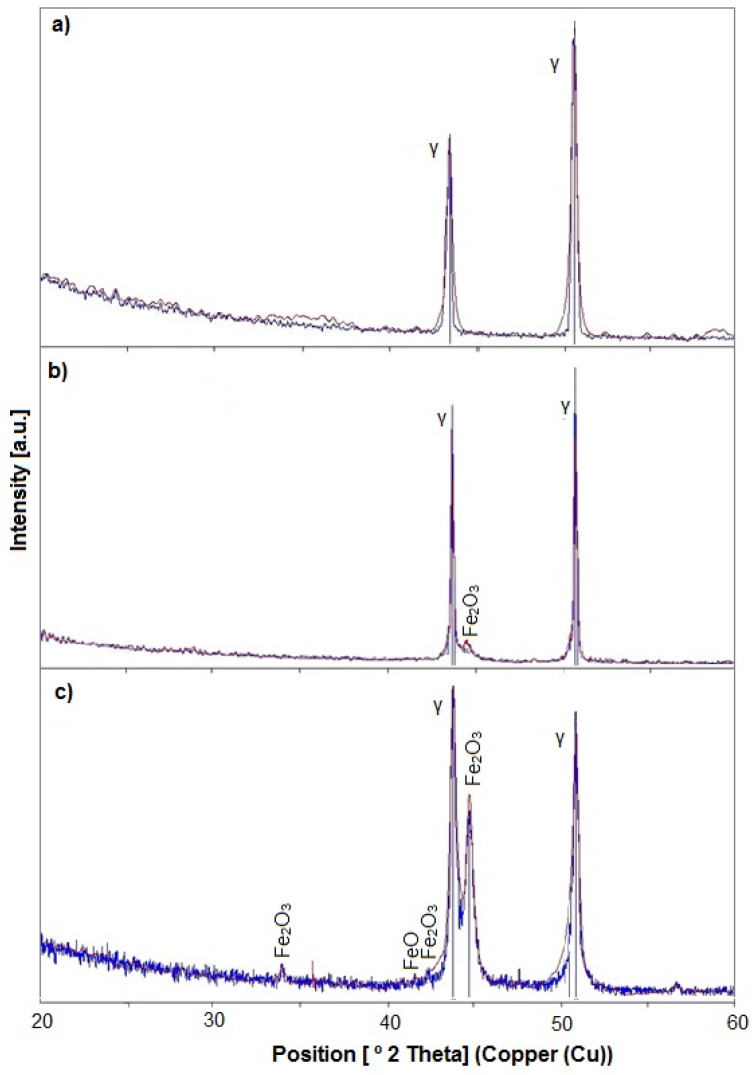
XRD spectrum for: (**a**) samples I, (**b**) samples II, (**c**) samples III.

Figure 6 shows the tested samples’ diffractograms. Reflections corresponding to the substrate were obtained for sample I and marked as ɣ. On the other hand, for samples II and III, the intensity of the γ peaks slightly decreased with the increase of the cut-off voltage, which is probably related to the growth of the porous layer. The measurement results show additional reflections which indicate the share of iron oxides (Fe_2_O_3_ and FeO) as a part of the modified surface. The presence of these oxides is visualized in the form of peaks that can be predicted for the 2-theta angles: 44.63°–Fe_2_O_3_, and 41.81°–FeO. The reflection corresponding to the 44.63° 2-theta angle is very clear and confirms the XPS findings regarding the significant presence of Fe_2_O_3_ oxide. The intensity of these peaks increased with the increase of the boundary voltage and is probably associated with the strength of the oxidation reaction [64].

### 3.2. Nanomechanical Properties

**Figure 7:**
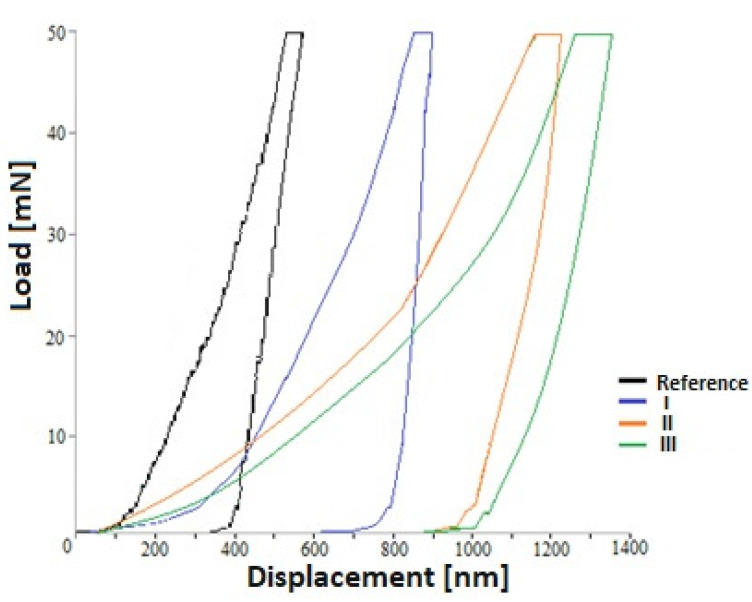
Hysteresis plots of load-deformation for a single indentation measurement.

Figure 7 shows the dependence of displacement into the surface on load. The values of nanohardness (H) and Young’s modulus (E) of the samples are presented in Table 6. When analyzing the test results, it can be noticed that with the increase of the voltage of the oxidation process, there was an apparent decrease in both H and E values for samples with the modified layer. The results of the research may be related to the structure of the formed layers and the size of the pores. The larger the pore diameter, the lower the H and E values. The surface quality had a significant influence on the results of the nanoindentation measurements. SEM tests for sample I showed areas where pores exist, but also areas on the surface on which the formation of the porous layer has started. Such areas covered a significant part of the sample surface. Perhaps some of the measurements of nanoindentation were made in these places, which would be suggested by the values closest to the results of the nanoindentation measurements carried out on the base material.

Surface roughness could induce underestimation of E and H at shallow indentation depth (i.e., small load) [65,66]. The highest values of the Young’s modulus were obtained for 316L and sample I, which at the same time revealed the lowest value of layer thickness, with a heterogeneous structure of the layer and the largest pore diameter.

One of the preliminary bioactivity assessment methods is surface wettability, which is characterized by the contact angle, affecting the degree of adsorption and aggregation of the material. It is related to the physical phenomena occurring on their surface, mainly the surface energy, defined by positively or negatively charged functional groups [64]. It is known [67] that cells do not adhere directly to the surface of the biomaterial, but through a layer of absorbed proteins which become attracted to the surface due to the interaction of electrostatic and van der Waals forces. Cell membrane receptors recognize proteins and activate intracellular receptors, leading to osteoblast adhesion to the implant surface. The adhered cells proliferate and differentiate, leading to the formation of new bone tissue. The degree and time at which the implanted material absorbs moisture from the environment of the human body has a strong influence on protein adsorption and cell adhesion [64] and determines plaque aggregation, water absorption, as well as the hydrophilicity or hydrophobicity of a given material.

The conducted tests (Table 7) showed that the Ti13Nb13Zr titanium alloy exhibits hydrophobic properties (contact angle value 81°), while the titanium oxide layer obtained on the titanium alloy has better hydrophilic properties compared to the pure alloy (contact angle 76°). On the other hand, the increase in the LVMAO anodizing voltage caused the contact angle to drop down to 56° (for sample III). It is known that the best value of the contact angle for attaching cells to the implant was determined to be 55° [68,69], while the most desirable value of the contact angle for hard tissue regeneration is 35–80° [22,68,70]. Such a low contact angle enabled the efficient adhesion of cells, proteins, and microorganisms on the surface of the implantable material. Its presence can be attributed to the porous structure of the surface. It is known that a low value of the contact angle results in good bioactivity, which enables the adhesion of cells and microorganisms on the surface of the implanted material. Such values of the contact angle accelerate the process of adhesion of the adhesive serum protein to cells, namely fibronectin and vitronectin [69], and such values of the contact angle were obtained. Excessively hydrophobic surfaces enhance cell affinity and reduce biocompatibility, but highly hydrophilic surfaces prevent cell–cell interactions, which are particularly important in tissue engineering [70,71].

**Figure 8:**
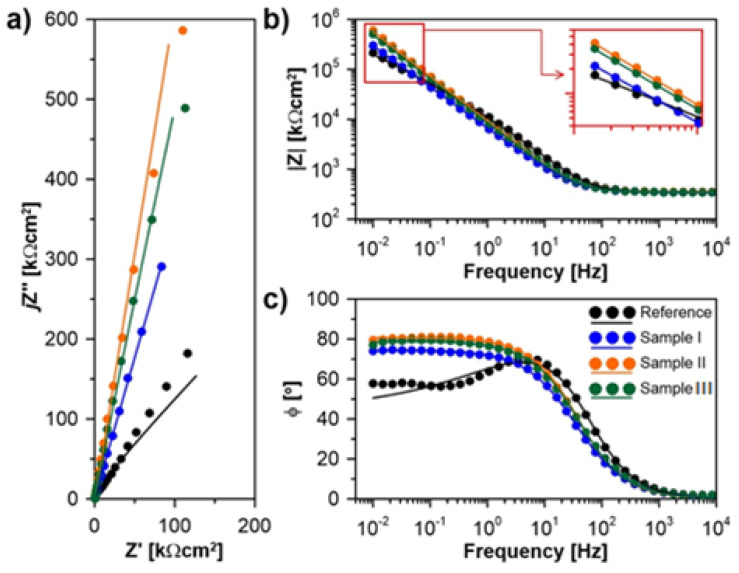
The EIS spectra recorded at open circuit potential conditions for each studied sample exposed to Ringer solution at 37 °C, after 2 h conditioning, presented in the form of: (**a**) Nyquist plot, (**b**,**c**) Bode plots of impedance modulus and phase angle, respectively. Experimental results are marked with points and EEC fitting results with solid line.

Finally, the electrochemical impedance spectroscopy (EIS) measurements were performed in order to determine the corrosion resistance of the stainless steels samples obtained by different surface modification protocols. It is one of the most often used techniques to evaluate the corrosion resistance of materials [72,73] in particular for passive films and coatings, where polarization curves analysis may be burdened by high errors. The measurements were carried out in Ringer’s solution, at 37 °C degrees, to imitate the human body fluids environment. The results of the corrosion resistance test are presented in Figure 8 and Table 8.

The shape of the impedance spectra shows very high resistance recorded for each studied stainless-steel sample, with the polarization resistance range in hundreds of kΩ. These are well observed in the Nyquist plot (Figure 6a) and the low-frequency range of the Bode impedance modulus plot (Figure 6b, inset). The impedimetric analysis confirms the XPS observation regarding the higher nobility of samples with formed protective porous layers, leading to the significantly increased corrosion resistance of these samples.

In order to provide a more detailed explanation, an electric equivalent circuit (EEC) should be selected. Here, we used a classic Randles circuit, modified by using the constant phase element (CPE) instead of the capacitor. The EEC is composed of the series resistance R_S_ (electrolyte resistance) and a parallel connection of the resistor *R_P_* and CPE, responsible for the charge transfer and accumulation processes occurring at the solid/liquid interface and the passive layer. The role of the CPE is to take into consideration the heterogeneity introduced by surface geometry factors, but also intermetallic phases, steel polycrystallinity, and others [64,65,72]. The impedance *Z_CPE_* may be described using Equation (1).

(1) $$Z_{CPE}=\frac{1}{Q{\left(j\omega \right)}^{\alpha }}$$

where *j* is an imaginary number, *ω* is the angular frequency, *Q* is the quasi-capacitance of the heterogeneous sample, and *α* is the CPE exponent. Notably, if *α* = 1, the CPE describes the ideal capacitor with capacitance Q. Thus, *α* is often called the heterogeneity factor. The EEC fit quality in a wide frequency range is given by the comparison of the experimental electric parameter values (Figure 8, points) versus fitted values (Figure 8, solid line). The EEC fitting results are summarized in Table 8.

Interestingly, regardless of the porosity of the anodized layer, each sample subjected to near-MAO anodic voltage seems to be characterized by higher electric homogeneity. The *α* parameter rises from 0.76 for the reference sample to 0.88 and 0.90 for Sample II and Sample III, respectively. These two samples are also characterized with the highest value of the parallel resistance *R_P_*, which is inversely proportional to the corrosion rate of the investigated steel. However, even for Sample I, which showed the lowest corrosion resistance among all studied layers, the *R_P_* value increased by a factor of 46. A terrific corrosion protection efficiency was noted for each studied coating, ranging from 97.9% for Sample I to 99.1% and 98.6% for Samples II and III, respectively. This parameter was adapted from corrosion inhibitor efficiency studies [72,74] and can be estimated using Equation (2).

(2) $$Prot.eff=\left(1-\frac{R_{P}^{reference}}{R_{P}^{coating}}\right)$$

## 4. Conclusions

The aim of the work was the modification the surface of 316L steel, which would be characterized by a developed surface topography, a given porosity, and a specific chemical composition. It has been shown that with the use of low voltage micro-arc oxidation (LVMAO), it is possible to produce porous layers, the chemical composition of which depends on the chemical composition of the parent material and the electrolyte used.

An increase in the anodization process voltage causes an increase in the thickness of the layer (almost six times) and changes the size of the pores present in the layer as well as the amount of elements introduced from the solution. At the same time, the tests of the layers show that, as a result of the use of a lower current voltage (100 V) during the oxidation process, thin layers were obtained, the surfaces of which are not uniform, consisting of areas with a porous structure as well as a slightly modified surface, reflecting the initial stage of the formation of a porous layer. The processes taking place could be considered as standard anodization, during which current peaks are visible, which indicates dissolution of the surface and repeated repassivation. After exceeding the critical voltage, a breakdown of the passive layer is noticed.

The increase in tension (200 V) affects the structure of the created porous layer, such that evenly spaced pores are visible.

The formed oxides and the layers are very tight, which hinders the diffusion of Fe ions, thus protecting them from further oxidation and contributing to an increase in corrosion resistance. The created porous layer is a specific barrier preventing the penetration of environmental factors into the implant core, as well as the diffusion of the alloy elements to penetrate into the tissues surrounding the implant.

Unfortunately, the porous structure of the layer affects the strength properties of the material. It has been observed that the porous structure of the layers slightly reduces the properties of the layer, mainly H and E, which affect the wear processes of the layers and indicate susceptibility to plastic deformation. The increase in the voltage used during the electrochemical oxidation process reduces the value of Young’s modulus, the values of which are close to the value of the Young’s modulus of bone, which has a positive effect.

The hydrophilic surfaces of all samples ensure better cell adhesion and protein adsorption, and contribute to faster integration of the implant into the surrounding tissue.

Based on the test results obtained, the best solution is found with layers made on sample III. The proposed surface modification for various medical applications provides the best properties. Introducing an additional modification of the process consisting in the implementation of, among others, nanometals or pharmaceuticals may increase the interest in this type of solution and open up new possibilities in implantology.

## Figures and Tables

**Table 1:** Chemical composition of the 316L alloy, wt. pct. (according to the manufacturer’s certificate, Bibus Metals Ltd., Dabrowa, Poland).

C	Cr	Ni	Mo	Mn	Si	N	Fe
0.03	17.64	11.97	2.15	1.71	0.71	0.053	bal.

**Table 2:** The labels of electrochemical oxidation layers formed at process conditions.

Sample	Voltage [V]	Time [Min]
I	100	15
II	150	15
III	200	15

**Table 3:** Thickness of the layers after LVMAO with difference potencial.

Sample	Thickness [μm]
I	2.3–2.8
II	6.0–7.9
III	14.3–15.8

**Table 4:** EDS examinations of tested specimens on the samples cross-sections (in wt.%).

Element	Reference	Sample I	Sample II	Sample III
Fe	68.88	68.58	63.75	58.89
Cr	16.24	14.49	15.13	15.71
Ni	10.72	8.96	9.62	10.38
Mo	2.13	2.11	2.09	2.01
Mn	1.47	1.46	1.47	1.45
Si	0.56	0.54	0.57	0.58
O *	-	1.86	7.37	10.98

(*) the quantities must be regarded as only informative.

**Table 5:** The share of metallic elements and their chemical states, based on the results of high-resolution XPS analysis of the studied 316L samples.

Chemical State	*Fe 2p_3/2_*			*Cr 2p_3/2_*			*Mo 3d_7/2_*		*Ni 2p_3/2_*	
	Fe^0^	Fe_2_O_3_	Fe(OH)_3_	Cr^0^	Cr_2_O_3_	Cr(OH)_3_	Mo^0^	MoO_3_	Ni^0^	Ni(OH)_2_
BE [eV]	707.0	710.3	712.3	573.9	576.0	577.3	227.7	232.3	852.8	855.4
Reference	2.9	39.2	27.1	2.2	10.5	13.8	0.8	1.4	2.1	0.0
Sample I	6.0	27.2	19.1	3.5	16.1	17.7	1.4	3.3	2.3	3.4
Sample II	5.6	29.2	17.5	2.3	14.8	19.8	1.3	3.7	2.1	3.7
Sample III	5.9	26.1	17.8	3.1	12.9	22.6	1.9	4.6	2.2	2.9

**Table 6:** Mechanical properties of the tested specimens.

Sample	Max. Depth (nm)	Plastic Depth (nm)	Hardness (GPa)	Young’s Modulus (GPa)
Reference	214 ± 15	198 ± 4	2.89 ± 0.05	43.48 ± 0.23
I	83 ± 31	79 ± 3	2.26 ± 0.16	16.90 ± 7.30
II	115 ± 30	107 ± 7	1.77 ± 0.21	15.75 ± 5.09
III	131 ± 22	127 ± 2	0.92 ±0.42	13.10 ± 4.63

**Table 7:** The contact angle for the water droplet for the tested specimens.

Sample	Average Contact Angle [°]
Reference	81.93 ± 2.60
I	76.74 ± 3.43
II	63.41 ± 5.57
III	56.40 ± 3.32

**Table 8:** Electrochemical impedance spectroscopy (EIS) results of the studied porous layers, estimated using selected EEC.

	*R_S_* [Ωcm^2^]	*Q* [µFs^n−1^cm^−2^]	*α* [-]	*R_P_* [MΩcm^2^]	*Prot.eff* [%]
Reference	327	24.8	0.76	0.5	--
Sample I	341	33.2	0.83	23.2	97.9
Sample II	345	23.3	0.88	52.5	99.1
Sample III	352	20.8	0.90	34.4	98.6

## Data Availability

The data supporting reported results concerning those analyzed or generated during the study are available for inspection by the authors of the article.
